# Covid-19 vaccination and menstrual cycle length in the Apple Women’s Health Study

**DOI:** 10.1038/s41746-022-00711-9

**Published:** 2022-11-02

**Authors:** Elizabeth A. Gibson, Huichu Li, Victoria Fruh, Malaika Gabra, Gowtham Asokan, Anne Marie Z. Jukic, Donna D. Baird, Christine L. Curry, Tyler Fischer-Colbrie, Jukka-Pekka Onnela, Michelle A. Williams, Russ Hauser, Brent A. Coull, Shruthi Mahalingaiah

**Affiliations:** 1 Department of Biostatistics, Harvard T.H. Chan School of Public Health, Boston, MA USA; 2 Department of Environmental Health, Harvard T.H. Chan School of Public Health, Boston, MA USA; 3 Epidemiology Branch, National Institute of Environmental Health Sciences, Research Triangle Park, Durham, NC USA; 4 Health, Apple Inc., Cupertino, CA USA; 5 Department of Epidemiology, Harvard T.H. Chan School of Public Health, Boston, MA USA

**Keywords:** Reproductive signs and symptoms, Epidemiology

## Abstract

COVID-19 vaccination may be associated with change in menstrual cycle length following vaccination. We estimated covariate-adjusted differences in mean cycle length (MCL), measured in days, between pre-vaccination cycles, vaccination cycles, and post-vaccination cycles within vaccinated participants who met eligibility criteria in the Apple Women’s Health Study, a longitudinal mobile-application-based cohort of people in the U.S. with manually logged menstrual cycles. A total of 9652 participants (8486 vaccinated; 1166 unvaccinated) contributed 128,094 cycles (median = 10 cycles per participant; inter-quartile range: 4–22). Fifty-five percent of vaccinated participants received Pfizer-BioNTech’s mRNA vaccine, 37% received Moderna’s mRNA vaccine, and 8% received the Johnson & Johnson/Janssen (J&J) vaccine. COVID-19 vaccination was associated with a small increase in MCL for cycles in which participants received the first dose (0.50 days, 95% CI: 0.22, 0.78) and cycles in which participants received the second dose (0.39 days, 95% CI: 0.11, 0.67) of mRNA vaccines compared with pre-vaccination cycles. Cycles in which the single dose of J&J was administered were, on average, 1.26 days longer (95% CI: 0.45, 2.07) than pre-vaccination cycles. Post-vaccination cycles returned to average pre-vaccination length. Estimated follicular phase vaccination was associated with increased MCL in cycles in which participants received the first dose (0.97 days, 95% CI: 0.53, 1.42) or the second dose (1.43 days, 95% CI: 1.06, 1.80) of mRNA vaccines or the J&J dose (2.27 days, 95% CI: 1.04, 3.50), compared with pre-vaccination cycles. Menstrual cycle change following COVID-19 vaccination appears small and temporary and should not discourage individuals from becoming vaccinated.

## Introduction

While research on the COVID-19 vaccine and menstrual characteristics is limited, existing studies have reported temporarily increased menstrual cycle length^1–3^, increased menstrual bleeding^4,5^, longer duration of menses^6^, and menstrual irregularities^7,8^, as well as breakthrough bleeding in individuals who previously menstruated^5^. Generally, these changes were considered short term, but little follow-up data have been presented. These reports suggest the possibility of temporary changes in menstrual cycles following COVID-19 vaccination^9,10^.

Though the United States Vaccine Adverse Event Reporting System (VAERS) does not actively collect information regarding menstrual cycles, over 11,000 people self-reported a menstrual-related issue to the system following COVID-19 vaccination by April 2022^11^. Events included heavy bleeding, irregular or delayed menstruation, oligomenorrhea, and amenorrhea. Likewise, the United Kingdom’s Medicines and Healthcare Products Regulatory Agency (MHRA) yellow card surveillance scheme received over 50,000 reports of menstrual disorders, including heavier than usual periods, delayed periods, and unexpected bleeding, after the COVID-19 vaccine^12^. This anecdotal evidence suggested the possibility of temporary changes in menstrual cycles following COVID-19 vaccination^9,10^. Individuals reported these menstrual changes to their doctors and posted about them on social media^13,14^. Various news outlets publicized the anecdotal reports^15–17^.

COVID-19 vaccination is widely regarded as safe^18,19^, and there is no evidence that it affects fertility in laboratory models^20,21^, clinical trials^22,23^, or observational studies^24–26^. Still, the immune response or the associated stress may link the vaccine with certain temporary menstrual changes^9^. Improved understanding of these associations, such as whether one exists and the magnitude and persistence of potential changes, will assist clinicians in counseling patients concerning vaccination^27^.

Here, we evaluate the relationship between COVID-19 vaccination and menstrual cycle length over time in the Apple Women’s Health Study (AWHS), a longitudinal digital cohort of people in the U.S. with manually logged menstrual cycles^28^. We compare pre-vaccination cycle lengths with those in which a vaccine dose was administered and cycles following vaccination.

## Results

A total of 257,838 menstrual cycles from 15,001 participants who completed the COVID-19 Vaccine Update survey and tracked at least one cycle, regardless of vaccination status or timing, were initially identified. After applying the remaining exclusion criteria, 128,094 menstrual cycles from 9652 participants were included in the current analysis (Supplementary Fig. 1). Participants recorded 13 cycles on average [median = 10; inter-quartile range (IQR): 4–22 (Supplementary Fig. 2 and Supplementary Note 1)]. The average within-individual standard deviation in cycle length was 4.2 days. Eighty-eight percent of participants were vaccinated; 12% were not. Fifty-five percent of vaccinated participants received the Pfizer-BioNTech vaccine (*N* =  4625), 37% received Moderna (*N* = 3129), and 8% received J&J (*N* = 676). For cycles in which a dose was received, 3410 participants received dose 1 (2.7% of all cycles), 3454 received dose 2 (2.7%), and 337 received the J&J dose (0.3%). A total of 7490 long cycles were identified, 6% of all cycles included. Vaccinated participants tended to be in the older age groups [*N* = 2526 (30%) vaccinated > 40 years old, *N* = 242 (21%) unvaccinated > 40 years old], to have a college or graduate degree [*N* = 5614 (66%) vaccinated, *N* = 284 (24%) unvaccinated], to be married [*N* = 3999 (47%) vaccinated, *N* = 433 (37%) unvaccinated], and to be nulliparous [*N* = 5101 (60%) vaccinated, *N* = 512 (44%) unvaccinated (Table 1 and Supplementary Table 1)]. The distribution of BMI and race/ethnicity were similar between vaccinated and unvaccinated participants.

**Table 1:** Demographic characteristics among 9652 participants in the Apple Women’s Health Study at enrollment and number of cycles included among 128,094 total cycles.

		Vaccinated		Unvaccinated		Overall	
		Participants	Cycles	Participants	Cycles	Participants	Cycles
		(*N*_p_ = 8486)	(*N*_c_ = 113,890)	(*N*_p_ = 1166)	(*N*_c_ = 14,204)	(*N*_p_ = 9652)	(*N*_c_ = 128,094)
Age	<20	240 (2.8)	3210 (2.8)	58 (5.0)	672 (4.7)	298 (3.1)	3882 (3.0)
	20–24	1038 (12.2)	11,843 (10.4)	168 (14.4)	1724 (12.1)	1206 (12.5)	13,567 (10.6)
	25–29	1475 (17.4)	16,907 (14.8)	220 (18.9)	2294 (16.2)	1695 (17.6)	19,201 (15.0)
	30–34	1601 (18.9)	19,345 (17.0)	259 (22.2)	2974 (20.9)	1860 (19.3)	22,319 (17.4)
	35–39	1606 (18.9)	22,454 (19.7)	219 (18.8)	2904 (20.4)	1825 (18.9)	25,358 (19.8)
	40–44	1361 (16.0)	21,619 (19.0)	144 (12.3)	2172 (15.3)	1505 (15.6)	23,791 (18.6)
	45–49	848 (10.0)	14,175 (12.4)	75 (6.4)	1137 (8.0)	923 (9.6)	15,312 (12.0)
	≥50	317 (3.7)	4337 (3.8)	23 (2.0)	327 (2.3)	340 (3.5)	4664 (3.6)
Race/Ethnicity	White, non-Hispanic	6276 (74.0)	84,242 (74.0)	848 (72.7)	10,334 (72.8)	7124 (73.8)	94,576 (73.8)
	Hispanic/Latina	910 (10.7)	11,651 (10.2)	137 (11.7)	1559 (11.0)	1047 (10.8)	13,210 (10.3)
	Other	1300 (15.3)	17,997 (15.8)	181 (15.5)	2311 (16.3)	1481 (15.3)	20,308 (15.9)
BMI	Underweight	139 (1.6)	1880 (1.7)	43 (3.7)	587 (4.1)	182 (1.9)	2467 (1.9)
	Normal weight	2899 (34.2)	39,840 (35.0)	355 (30.4)	4474 (31.5)	3254 (33.7)	44,314 (34.6)
	Overweigh	2192 (25.8)	28,828 (25.3)	300 (25.7)	3659 (25.8)	2492 (25.8)	32,487 (25.4)
	Obese	3108 (36.6)	39,800 (34.9)	450 (38.6)	5263 (37.1)	3558 (36.9)	45,063 (35.2)
	Missing	148 (1.7)	3542 (3.1)	18 (1.5)	221 (1.6)	166 (1.7)	3763 (2.9)
Parity	Parous	3335 (39.3)	45,251 (39.7)	643 (55.1)	7951 (56.0)	3978 (41.2)	53,202 (41.5)
	Nulliparous	5101 (60.1)	67,986 (59.7)	512 (43.9)	6200 (43.6)	5613 (58.2)	74,186 (57.9)
	Missing	50 (0.6)	653 (0.6)	11 (0.9)	53 (0.4)	61 (0.6)	706 (0.6)

Values are presented as *N* (%) of total participants included in the analysis. *N*_p_ = number of participants. *N*_c_ = number of cycles. Other race/ethnicity includes Black, non-Hispanic (African American or African), Asian, American Indian or Alaska Native, Middle Eastern or North African, Native Hawaiian or Pacific Islander, more than one race/ethnicity, or participants who reported that none of the available categories fully described them. *BMI* body mass index; underweight; BMI < 18.5 kg/m^2^; normal: 18.5 ≤ BMI < 25 kg/m^2^, overweight: 25 ≤ BMI < 30 kg/m^2^, Obese: BMI ≥ 30 kg/m^2^.

### Change in menstrual cycle length

Using covariate-adjusted conditional linear regression, we found menstrual cycles when participants received the vaccine to be, on average, longer than their pre-vaccination cycles (Table 2). Among vaccinated participants, COVID-19 vaccination was associated with a small increase in length for cycles when participants received the first dose (0.50 days, 95% CI: 0.22, 0.78) and cycles when participants received the second dose (0.39 days, 95% CI: 0.11, 0.67) of mRNA vaccines compared with pre-vaccination cycles. Cycles in which the single dose of J&J was administered were, on average, 1.26 days longer (95% CI: 0.45, 2.07) than pre-vaccination cycles. Post-vaccination cycles returned to average pre-vaccination length, with 0.14 days (95% CI: –0.13, 0.40) in the first cycle following vaccination, 0.13 days (95% CI: –0.14, 0.40) in the second cycle, –0.17 days (95% CI: –0.43, 0.10) in the third cycle, and –0.25 days (95% CI: –0.52, 0.01) in the fourth cycle.

**Table 2:** Adjusted within-participant change in mean menstrual cycle length and 95% confidence intervals (95% CIs) and adjusted odds ratios (ORs) and 95% CIs of experiencing a long menstrual cycle (>38 days) comparing cycles in which a vaccine was administered and post-vaccination cycles with pre-vaccination cycles.

	Difference in mean cycle length (95% CI)	Odds ratio of having a long cycle (95% CI)
Pre-vaccination cycles	Reference	Reference
First dose	0.50 (0.22, 0.78)	1.13 (0.87, 1.48)
Second dose	0.39 (0.11, 0.67)	1.08 (0.83, 1.43)
J&J dose	1.26 (0.45, 2.07)	2.16 (1.16, 4.03)
Post-vaccination cycles		
First cycle	0.14 (−0.13, 0.40)	0.94 (0.73, 1.21)
Second cycle	0.13 (−0.14, 0.40)	0.95 (0.74, 1.22)
Third cycle	−0.17 (−0.43, 0.10)	0.76 (0.59, 0.99)
Fourth cycle	−0.25 (−0.52, 0.01)	0.71 (0.55, 0.93)

Differences are from conditional linear regression. Odds ratios are from conditional logistic regression. The conditional models control for all participant-level characteristics; models are additionally adjusted for age, BMI, and seasonality. Doses 1 and 2 were Pfizer-BioNTech or Moderna; *J&J* Johnson & Johnson/Janssen.

The conditional logistic regression model of the probability of a long cycle suggested that, compared with pre-vaccination cycles, participants were more likely to experience a long cycle during the cycle in which they received the J&J vaccine [OR = 2.16, 95% CI: 1.16, 4.03 (Table 2 and Supplementary Fig. 3)]. There was no evidence of increased probability of a long cycle for cycles in which participants received the first dose (OR = 1.13, 95% CI: 0.87, 1.48) or the second dose (OR = 1.08, 95% CI: 0.83, 1.43) of mRNA vaccines. There was, likewise, no evidence of increased odds of long cycles in the first cycle (OR = 0.94, 95% CI: 0.73, 1.21) or the second cycle (OR = 0.95, 95% CI: 0.74, 1.22) post-vaccination, regardless of the vaccine type. In the third and fourth post-vaccination cycles, the odds of a long cycle decreased, with OR = 0.76 (95% CI: 0.59, 0.99) in the third cycle and OR = 0.71 (95% CI: 0.55, 0.93) in the fourth cycle following the single J&J dose or the second dose of an mRNA vaccine.

### Effect modification by menstrual cycle phase

The association between vaccine dose and mean cycle length depended on the phase in which the dose was received (Table 3). Compared with pre-vaccination cycles, follicular phase vaccination with an mRNA vaccine was associated with an increase in mean cycle length in first-dose cycles (0.97 days, 95% CI: 0.53, 1.42) or second-dose cycles (1.43 days, 95% CI: 1.06, 1.80). For the J&J dose, follicular phase vaccination was associated with a 2.27 (95% CI: 1.04, 3.50) day increase in cycle length. There was no evidence of increased mean cycle length in cycles in which the first mRNA vaccine dose (0.21 days, 95% CI: –0.14, 0.57) or the J&J vaccine dose (0.39 days, 95% CI: –0.75, 1.53) was administered in luteal phase. However, luteal phase vaccination was associated with a decrease in average length for cycles in which the second mRNA dose was administered (–0.97 days, 95% CI: –1.39, –0.55), compared with pre-vaccination cycles. Regression coefficients for post-vaccination cycles did not meaningfully change with the inclusion of cycle phase.

**Table 3:** Adjusted within-participant change in mean menstrual cycle length and 95% confidence intervals (95% CIs) comparing cycles in which a vaccine was administered and post-vaccination cycles with pre-vaccination cycles by menstrual cycle phase of vaccine dose.

	Difference in mean cycle length (95% CI)	Difference from coefficient for dose in luteal phase (95% CI)
Pre-vaccination cycles	Reference	–
Dose in follicular phase		
First dose	0.97 (0.53, 1.42)	0.76 (0.16, 1.35)
Second dose	1.43 (1.06, 1.80)	2.40 (1.81, 2.99)
J&J dose	2.27 (1.04, 3.50)	1.89 (0.20, 3.57)
Dose in luteal phase		
First dose	0.21 (−0.14, 0.57)	Reference
Second dose	−0.97 (−1.39, -0.55)	Reference
J&J dose	0.39 (−0.75, 1.53)	Reference
Post-vaccination cycles		
First cycle	0.16 (−0.11, 0.44)	---
Second cycle	0.15 (−0.12, 0.43)	---
Third cycle	−0.14 (−0.42, 0.13)	---
Fourth cycle	−0.24 (−0.51, 0.04)	---

Differences are from conditional linear regression. Differences between regression coefficients are for a given dose in the follicular vs. luteal phase. The conditional model controls for all participant-level characteristics; models are additionally adjusted for age, BMI, and seasonality. Doses 1 and 2 were Pfizer-BioNTech or Moderna; *J&J* Johnson & Johnson/Janssen.

In the covariate-adjusted mixed-effects model, we found no evidence of a difference between mean menstrual cycle length in vaccinated and unvaccinated participants (0.24 days, 95% CI: –0.34, 0.82). In the GEE, the probability of a long cycle was slightly greater in unvaccinated than in vaccinated participants (OR = 1.20, 95% CI: 1.00, 1.44). Regression coefficients for vaccine doses were similar to but larger in magnitude than those from conditional regression models (Supplementary Tables 2 and 3).

### Sensitivity analyses

Fifty-eight percent of participants were included in the sensitivity analysis restricted to those with three or more tracked cycles. While 82% of participants had three or more tracked cycles, only 53% of vaccinated participants had two or more tracked cycles before vaccination and at least one tracked cycle in which a vaccine was administered or after vaccination. Three percent of cycles had missing values and were dropped in the complete case analysis. Ninety-seven percent of cycles were tracked before completion of the COVID-19 Vaccine Update survey, and 93% of participants who had three or more tracked cycles had an average cycle length of 24–38 days. Fifty-three percent of participants reported that they had never tested positive for COVID-19, 24% reported that they had tested positive for COVID-19, and the remaining 23% did not complete the updated COVID-19 Vaccine Update survey. Results from sensitivity analyses were consistent with those from the main analyses (Supplementary Tables 4–6). When participants who received two doses in a single menstrual cycle were included, these cycles were, on average, 4.96 days (95% CI: 4.42, 5.50) longer than pre-vaccination cycles, and cycles between doses were, on average, 1.94 days shorter (95% CI: –2.65, –1.23) than pre-vaccination cycles (Supplementary Table 7). When including estimated cycle phase of vaccination in the analysis, two-dose cycles in which the first dose was received early to mid-cycle, roughly corresponding with the follicular phase, were similarly long, on average, compared with pre-vaccination cycles (Supplementary Table 8).

## Discussion

In this large prospective cohort of participants in the U.S., we examined differences in menstrual cycle length in relation to the type of COVID-19 vaccine administered and the timing of administration. Our results suggest a small, non-persistent increase in average length of menstrual cycles in which a vaccine was administered. Moreover, the J&J vaccine was associated with a significant increase in the probability of a clinically long (>38 days) cycle; however, this increase did not persist over time. We found evidence that vaccination increased cycle length for approximately 1–2 cycles post-vaccination. After this, cycles returned to their pre-vaccination mean. We saw that doses received early to mid-cycle, roughly corresponding with the follicular phase, explained the increase in mean cycle length observed in the primary results. We also observed that when a second dose was received in the last 14 days of a cycle, roughly corresponding with the luteal phase, that cycle was, on average, shorter. Together, these results indicate that the association between vaccination and cycle length differs by the timing of the dose’s administration, potentially corresponding with cycle phase.

Previous studies have shown increased cycle length^1^, as well as menstrual cycle irregularities in relation to COVID-19 vaccination^4,6–8^. The current work is consistent with results from Edelman et al. and Alvergne et al. that showed a small increase in length during cycles when vaccination occurred^1,2^. We extended this work to evaluate vaccine timing and menstrual cycle length and found follicular dosing of all vaccines associated with longer cycles, while a second dose of an mRNA vaccine in the luteal phase was associated with slightly shorter cycles.

Potential mechanisms underlying the change in menstrual cycle length may involve inflammation from the immune response to vaccination. This immune response may impact (a) signaling between the hypothalamus, pituitary, and ovaries (HPO), resulting in (i) prolongation of follicular recruitment and, as a result, elongation of menstrual cycle length^29^, or (ii) suppression of the growth of the endometrial lining^30^, and (b) endometrial stability in the luteal phase, causing a reduction in cycle length^31,32^.

The onset of menstruation is characterized by endometrial breakdown and apoptosis. Immune cells derived from menstrual blood demonstrate production of inflammatory markers (e.g., interferon (IFN)-gamma, granzyme B, and perforin)^33^, and genes related to inflammatory processes (e.g., lipopolysaccharide binding protein, SERPINB3, SERPINB4, IL17C, and SPINK1) are up-regulated^34^. mRNA vaccines may potentially trigger an innate immune response by activating pro-inflammatory cytokines, type I IFNs, and pro-inflammatory genes^35^. In this way, the Pfizer-BioNTech and Moderna vaccines may disturb the menstrual cycle by promoting local and systemic inflammation.

Individual cycle length varies naturally^36,37^, and the International Federation of Gynecology and Obstetrics classifies a difference of less than 8 days between an individual’s shortest and longest cycles as normal^38^. The magnitude of the change we see in our data following vaccination is well within this natural variability. In contrast, infection by the COVID-19 virus may cause potentially long-term disruption of menstrual function^39–44^, though recent findings from the Nurses’ Health Study 3 found no association between COVID-19 infection and changes in usual menstrual cycle characteristics^3^. Research has found no evidence that COVID-19 vaccination adversely impacts fertility outcomes^20–26^. COVID-19 vaccination is widely regarded as safe for people who menstruate^45,46^, and this work supports that conclusion.

This study has several strengths. First, a large sample size (128,094 menstrual cycles from 9652 participants), allowed for sufficient statistical power to detect minor differences measured in fractions of days. Further, the AWHS includes a diverse population across a wide range of demographic characteristics (e.g., age, race/ethnicity, education, BMI category). Moreover, the AWHS is not limited to users of a specific cycle tracking app, which may target certain demographic groups; instead, cycle tracking can occur directly through the Health app or through a variety of third-party apps. Additionally, menstrual cycle characteristics were reported prospectively. We used multivariable models to adjust for potential confounding in all analyses. In our main analyses, we used conditional, fixed effect models, which control for individual-level features. This removed any avenue for confounding by sociodemographic factors or differential tracking behavior across participants. Finally, we controlled for seasonal variation in the outcome that is independent of vaccination status (i.e., exhibited by all participants in the study) by including an indicator variable for month and year of cycle; this further accounted for pandemic era experiences and shared social stressors in both vaccinated and unvaccinated participants.

Our findings should be interpreted in light of several limitations. First, because our analysis considered mean cycle length in the population, it cannot distinguish subpopulations, such as participants with preexisting conditions, who may have experienced more extreme changes in cycle length. Second, we relied on self-report for menstrual characteristics, COVID-19 vaccination details, and reported covariates. Consequently, the accuracy of the estimated menstrual cycle length may be affected by user tracking behavior, on which we have limited information. However, use of conditional models for our primary analyses controlled for differences in tracking behavior between participants, and the precise indicator variable for month and year controlled for potential changes in tracking behavior over time, leaving the only path for temporal confounding through differential changes in tracking behavior over time by treatment (i.e., in vaccinated and unvaccinated participants). An additional limitation is the possibility of length-time bias where the probability of receiving a vaccination is higher in longer cycles which may then bias the estimate for the association of cycle length with vaccination towards increased cycle length; therefore, our overall conclusions of no strong associations are robust to this bias. We also had limited data on menses and were thus unable to address potential associations between the COVID-19 vaccine and bleed length or flow characteristics. We lacked measurements for participants’ hormone levels to determine the follicular and luteal phases of a given cycle; instead, we calculated this using a standard approach, which could affect the phase-specific estimates and the comparisons between them^47,48^. As our study population only included iPhone users in the U.S., our results may not be generalizable to all U.S. individuals who menstruate or to other populations. Additionally, given available data, we did not include participants with two vaccine doses in a single cycle in our main analyses, and we did not report on cycles between doses. In a sensitivity analysis, cycles in which two doses were received appeared close to five days longer than pre-vaccination cycles, on average. However, cycles in which two vaccines were administered could not be shorter than the number of days between doses (usually 21 or 28 days), truncating the distribution of menstrual cycle length and making these cycles longer, by definition, and not necessarily a result of vaccination. Likewise, cycles between doses appeared close to two days shorter than pre-vaccination cycles, on average. However, cycles between doses could not be longer than the number of days between doses, truncating the distribution of menstrual cycle length and making these cycles shorter, by definition, and not necessarily a result of vaccination. As receiving two doses in a single cycle or having a cycle between doses was dependent on menstrual cycle length, we did not present these findings as main results, and further research is required to determine the direction of this relationship. It is possible that vaccination in the follicular phase extended cycle length in this subset of participants such that the second dose fell in the same cycle, and this relationship warrants further investigation. Finally, we cannot exclude the possibility of residual confounding from an unidentified time-varying factor. However, our findings were consistent after restricting to participants who reported never testing positive for COVID-19. Furthermore, we adjusted for multiple factors that varied within participant (and between participants in mixed-effects models) to account for confounding from multiple directions. We do not expect, therefore, that residual confounding can fully explain our results.

In the present study, we estimated the association between COVID-19 vaccination and menstrual cycle length in the cycles in which vaccination occurred and in succeeding cycles. Vaccination was associated with a very small increase in cycle length that was well within the natural variability in the study population. This change appeared driven by doses received in the follicular phase of the cycle. The magnitude of the increase diminished in each cycle following vaccination, and no association with cycle length persisted over time. This change appears minor and temporary and should not discourage individuals from becoming vaccinated.

## Methods

### Study design and population

The AWHS is a prospective digital cohort of participants in the U.S.. Recruitment began in November 2019 and is currently on-going^28^; the last recruitment date for this cohort was January 31, 2022. Enrollment eligibility specifies that participants must be assigned female at birth, have menstruated at least once, live in the U.S., be at least 18 years old (at least 19 years old in Alabama and Nebraska and at least 21 years old in Puerto Rico), be able to communicate through written and spoken English, have an iPhone with a compatible version of iOS, download the Apple Research app, be the sole user of an iPhone and an iCloud account, and provide written informed consent for participation. This study was approved by the Institutional Review Board at Advarra (CIRB #PRO00037562) and registered on Clinicaltrials.gov (NCT04196595).

Detailed information on study design, participant recruitment, and data collection has been described previously^28^. After enrollment, participants were surveyed monthly regarding their menstrual health. A COVID-19 Vaccine Update survey prompted participants to record their vaccination status and, if applicable, answer questions regarding their vaccine. For this analysis, we included eligible AWHS participants who completed the COVID-19 Vaccine Update survey and tracked at least one menstrual cycle.

### COVID-19 Vaccine Update survey and Cycle Tracking

The COVID-19 Vaccine Update survey was first delivered to participants in September 2021. This survey was redistributed quarterly or until participants completed it. It asked if participants received a COVID-19 vaccine, and they could respond “Yes,” “No,” or “Prefer not to answer.” Participants who answered “Yes” received follow-up questions to ascertain the date of the first and second (if applicable) vaccines, the type of first and second (if applicable) vaccines (Pfizer-BioNTech, Moderna, Johnson & Johnson/Janssen (J&J), or other), and any symptoms in the 48 h following a dose. Participants who completed the COVID-19 Vaccine Update survey with missing or incorrect dates [e.g., vaccination dates recorded before December 2020 (when vaccinations first became available) or after February 2022 (when this analysis was conducted) or second dose recorded before first dose] were excluded from this analysis. Questions concerning COVID-19 infection were not included in the original COVID-19 Vaccine Update survey, but they were added in January 2022; thus, for a subset of the study sample we have further information on infection. Dissemination of the COVID-19 Vaccine Update survey occurred prior to the authorization and roll out of additional doses and boosters in the U.S.^49^. Therefore, questions concerning the number of vaccines received was aligned with CDC classification of vaccination primary series^50^.

Characteristics of tracked menstrual cycles in the AWHS and methods for menstrual cycle identification have been described^51^. Participants logged menses (menstrual flow) and bleeding days, with associated dates, via Cycle Tracking in the Apple Health app or any third-party cycle tracking app that the participant permitted to share data as part of this study. These data could include logging collected prospectively after enrollment and entries from up to 2 years prior to enrollment^51^. We defined spotting as any bleeding that happens outside of the regular period, and we did not include it in this analysis. We defined the menstrual cycle as one or more consecutive days with tracked menstrual flow followed by two or more days of no tracked flow. We identified the first day of menstrual flow as the first day of the cycle, as defined in previous studies^52,53^. We excluded cycles shorter than ten days or longer than 90 days from the analysis because they were unlikely natural menstrual cycles (22). For the remaining cycles, we excluded cycles that were atypically long or likely artifacts due to gaps in record-keeping (i.e., skip-tracking) using participant-specific thresholds modified from a previous study with details in Li et al.^51,54^. The primary analysis included cycles tracked retrospectively as early as January 2018, and prospectively between enrollment and November 2021 when participants had not reported a hysterectomy and were not pregnant, lactating, menopausal, or using hormones.

For the current work, participants were categorized as vaccinated or unvaccinated as self-reported in the COVID-19 Vaccine Update survey (see Supplementary Table 9 for survey questions). Of the participants who responded “Prefer not to answer” concerning their vaccination status (*N* = 216) and participants who indicated that they had received one dose of a two-dose series (*N* = 257), none tracked menstrual cycles. Individual cycles for vaccinated participants were labeled as: (1) “pre-vaccination”, if the entire cycle occurred prior to the first vaccination, (2) “first dose,” cycle in which the participant received the first vaccine dose of the Pfizer-BioNTech or Moderna series (i.e., mRNA vaccines), (3) “second dose,” the cycle in which the participant received the second dose of an mRNA series, (4) “J&J dose,” the cycle in which the participant received the single Johnson & Johnson/Janssen dose, or (5) “post-vaccination,” with the number of cycles since vaccination (since the second dose of an mRNA series or since the single J&J dose, accordingly) recorded. Post-vaccine cycles 1–4 were assessed for potentially persistent associations over time; we limited analysis to four cycles post-vaccination because booster vaccines likely became available as early as cycle five. We categorized timing of vaccination within a cycle into early to mid-cycle, which approximates the follicular phase of the cycle, and late cycle, which approximates the luteal phase of the cycle to assess the impact of timing of the dose within a menstrual cycle, understanding that without an assessment of ovulation (to differentiate follicular from luteal phases), these categories are only proxies. For a given cycle beginning with the first bleed day, we defined the luteal phase as the 14 days prior to the start of the subsequent cycle, and we defined the follicular phase as the remaining days beginning with the first bleed day and ending on the day prior to the luteal phase^47,48^. Participants with a single cycle in which they received both doses were excluded from the current analysis due to the fact that these cycles could not be short, by definition, and their length was not necessarily a result of treatment.

### Covariates

Additional data were collected from survey questionnaires through the Apple Research app^28^. Final regression models included covariates that were a priori defined as potential confounders based on previous literature and a directed acyclic graph [(DAG) Supplementary Fig. 4]^55,56^. These include age, race/ethnicity, body mass index (BMI), parity, and season. We calculated participants’ age as the year of a given menstrual cycle minus their birth year. As previous studies have suggested a non-linear relationship between age and menstrual cycle length^47,57^, we categorized age as <20, 20–24, 25–29, 30–34, 35–39, 40–44, 45–49, and > 50. For this analysis, we divided race/ethnicity into three categories based on self-report at enrollment, White, non-Hispanic, Hispanic (Latina or Spanish), or other [including Black, non-Hispanic (African American or African), Asian, American Indian or Alaska Native, Middle Eastern or North African, Native Hawaiian or Pacific Islander, more than one race/ethnicity, or participants who reported that none of the available categories fully described them], due to the small number of participants in some groups. BMI was calculated from self-reported height and weight at enrollment, or as reported by participants when they were prompted to update these fields yearly; and the most recently recorded BMI per cycle was used. Participants were categorized as underweight (BMI < 18.5 kg/m^2^), normal (18.5 ≤ BMI < 25 kg/m^2^), overweight (25 ≤ BMI < 30 kg/m^2^), or obese (BMI ≥ 30 kg/m^2^). Parity was recorded as nulliparous or parous as reported in the reproductive history survey. Because of seasonal trends in menstrual cycle length and potentially irregular time trends due to shared experiences among all subjects (pandemic stress or other shared experiences)^58,59^, we choose to include a separate indicator for every month as a flexible means of controlling for season and time trends common to all subjects. While not as parsimonious as some commonly used methods, it provides more rigorous control of potential temporal confounding. Missingness was included as a category for all covariates.

### Statistical analysis

We examined distributional plots, contingency tables, and descriptive statistics for all variables of interest. To account for the longitudinal study design, we used linear regression models with subject-level fixed effects to estimate the covariate-adjusted strictly within-participant difference and 95% confidence intervals (95% CIs) in mean cycle length, measured in days, between pre-vaccination cycles and cycles in which a vaccine dose was administered and between pre-vaccination cycles and post-vaccination cycles^60^. We included participant ID as a fixed effect in the conditional model to account for longitudinal clustering of repeated measures within each participant. The fixed subject effect controls for all potential confounders that vary between participants. In this approach, participant-specific covariates that do not vary with time are not included in the model. This strategy is similar to a case-control study that includes matching of cases and controls on certain covariates. In the present analysis, the “matching” is within a participant and compares vaccinated and post-vaccine cycles (case cycles) to their own pre-vaccine cycles (control cycles)^60^. We defined long (>38 days) cycles using recommendations from the International Federation of Gynecology and Obstetrics and estimated the probability of experiencing a long menstrual cycle in cycles in which a vaccine was received, and post-vaccine cycles compared with pre-vaccine cycles^38^. To consider the chance of a long cycle in relation to vaccination, we fit a covariate-adjusted conditional logistic regression, which treats each participant as a matched set, and present odds ratios (ORs) and 95% CIs.

We further compared associations between vaccination and menstrual cycle length by the timing of vaccine dose within a menstrual cycle (i.e., early to mid-cycle, corresponding with the follicular phase, or late cycle corresponding with the luteal phase). Heterogeneity by menstrual cycle phase was considered by calculating the difference in effect estimates (e.g., first dose in the follicular phase versus first dose in the luteal phase) with the associated 95% CI; a 95% CI that did not contain zero was interpreted as a significant difference between the two coefficients.

As a secondary analysis, we used multivariable linear mixed-effects models with random participant-specific intercepts to estimate the covariate-adjusted difference and 95% CI in mean cycle length between vaccinated and unvaccinated participants. This was done because fixed effects regression does not provide estimates for independent variables that do not vary within participant (e.g., race/ethnicity and parity). Additionally, we used generalized estimating equations (GEE) to fit a logistic regression model that estimated ORs and 95% CI for the association between COVID-19 vaccination and the probability of having a long cycle in vaccinated participants compared with unvaccinated participants.

We conducted additional sensitivity analyses to assess the robustness of our results. First, we restricted analysis to participants who contributed at least three menstrual cycles; for vaccinated participants, we required at least two cycles before their first dose and at least one cycle in which a dose was administered or post-vaccination. Within participants who contributed three or more cycles, we calculated the average menstrual cycle length across all tracked cycles for unvaccinated participants and across pre-vaccination cycles for vaccinated participants and then restricted to those with an average cycle length of 24–38 days. We also retained data for participants who received two doses in a single cycle (*N* = 830) and cycles between doses (*N* = 510). Additionally, we restricted to participants who reported that they had not tested positive for COVID-19, and we conducted a complete case analysis by removing cycles with missing values for covariates. Finally, considering some participants may have reported being unvaccinated in the COVID-19 Vaccine Update survey but later received a vaccine, we restricted to cycles recorded prior to COVID-19 Vaccine Update survey completion.

Data management, processing, and statistical analyses were conducted in Python 3.6^61^. All statistical tests were two-sided. We present Bonferroni-adjusted 95% confidence intervals for all results, with *α* = 0.05/7 (i.e., 99.99% confidence intervals) for the main analysis to account for multiple comparisons among the seven main associations of interest (i.e., cycle length during first dose, second dose, J&J dose, and post-vaccination cycles 1–4, all compared with pre-vaccination cycles) because of the increased risk of a type I error.

### Reporting summary

Further information on research design is available in the Nature Research Reporting Summary linked to this article.

## Supplementary information

Supplemental material

Reporting Summary

## Data Availability

Aggregated data that support the findings of this study may be available upon request from the corresponding author [S.M.]. Any request for data will be evaluated and responded to in a manner consistent with policies intended to protect participant confidentiality and language in the Study protocol and informed consent form.

## Code availability

Code for all statistical analyses was written in Python 3.6. This code may be available upon request from the corresponding author [S.M.]. Any request for Python scripts will be evaluated and responded to in a manner consistent with policies intended to protect participant confidentiality and language in the Study protocol and informed consent form.
