# Supplementary material for: Covid-19 vaccination and menstrual cycle length in the Apple Women’s Health Study
Source: NPJ Digit Med. 2022 Nov 2;5:165. doi: 10.1038/s41746-022-00711-9 (PMC9628464; doi:10.1038/s41746-022-00711-9)

Supplemental Materials.

***Covid-19 vaccination and menstrual cycle length in the Apple Women’s Health Study***

Elizabeth A. Gibson,^1,+^ Huichu Li,^2,+^ Victoria Fruh,^2^ Malaika Gabra,^2^ Gowtham Asokan,^1^ Anne Marie Z. Jukic,^3^ Donna D. Baird,^3^ Christine L. Curry,^4^ Tyler Fischer-Colbrie,^4^ Jukka-Pekka Onnela,^1^ Michelle A. Williams,^5^ Russ Hauser,^2,5^ Brent A. Coull,^1,2^ Shruthi Mahalingaiah,^2,*^

+ These authors contributed equally

1 Department of Biostatistics, Harvard T.H. Chan School of Public Health, Boston, MA

2 Department of Environmental Health, Harvard T.H. Chan School of Public Health, Boston, MA

3 Epidemiology Branch, National Institute of Environmental Health Sciences, Research Triangle Park, North Carolina

4 Health, Apple Inc., Cupertino, CA

5 Department of Epidemiology, Harvard T.H. Chan School of Public Health, Boston, MA

* Corresponding author: shruthi@hsph.harvard.edu

**Figure legends**

**Supplementary Figure 1.** Flow chart of study identification, inclusion, and exclusion criteria for current analysis. N_p_ = number of participants. N_c_ = number of cycles.

**Supplementary Figure 2.** Histogram of number of cycles reported by participants in the Apple Women’s Health Study.

**Supplementary Figure 3.** Adjusted odds ratios (ORs) and 95% confidence intervals (CIs) of experiencing a long menstrual cycle (> 38 days) comparing cycles in which a vaccine was administered and post-vaccination cycles with pre-vaccination cycles. ORs are from conditional logistic regression model that controls for all participant-level characteristics; model additionally adjusted for age, BMI, and seasonality. First and second mRNA doses were Pfizer-BioNTech or Moderna; single dose J&J was Johnson & Johnson/Janssen. ORs are represented by points on the figure, and error bars represent the upper and lower bounds of the 95% CI.

**Supplementary Figure 4.** Directed Acyclic Graph (DAG) used to identify confounding variables as covariates for inclusion in regression models; Note: Figure generated using DAGitty v3.0.5. The green variable with the triangle, COVID-19 Vaccine, is the treatment, and the blue variable with the vertical bar, MCL, is the outcome of interest. The green arrow is the association of interest. Variables in red (race/ethnicity, SES, education, age, marital status, BMI, parity, and season) are identified as confounders, and red arrows are confounding paths. The variable in green, region, affects only the exposure, and the black arrow is a non-confounding path. This DAG identifies five variables—age, race/ethnicity, parity, BMI, and season—as the minimally sufficient adjustment set for estimating the total effect of the COVID-19 vaccine on MCL. Acronyms: SES = socioeconomic status; BMI = body mass index; MCL = mean cycle length.

Supplementary Notes

Supplementary Note 1. Participant cycle contribution

A total of 1,086 participants had only one cycle in the sample, and 698 had only two cycles. Participants with only one cycle did not contribute to the conditional regression models (main results) at all, and participants with two cycles only contributed to the conditional models if they had one reference cycle (i.e., pre-vaccination cycle) and one cycle in a treatment group (i.e., cycle with a dose or cycles 1-4 following the vaccine) (n = 376). Participants with one or two cycles were included in Table 1 and mixed-effects models (Supplementary Tables 2, 3, and 5).

Supplementary Tables

Supplementary Table 1. Additional demographic characteristics among 9,652 participants in the Apple Women’s Health Study at enrollment and number of cycles included among 128,094 total cycles. Values are presented as N (%) of total participants included in the analysis. N_p_ = number of participants. N_c_ = number of cycles.

|  | Vaccinated^a^ | | | | Unvaccinated^a^ | | | | Overall^a^ | | | |
| --- | --- | --- | --- | --- | --- | --- | --- | --- | --- | --- | --- | --- |
|  | Participants | | Cycles | | Participants | | Cycles | | Participants | | Cycles | |
|  | (N_p_ = 8,486)^b^ | | (N_c_ = 113,890)^b^ | | (N_p_ = 1,166)^b^ | | (N_c_ = 14,204)^b^ | | (N_p_ = 9,652)^b^ | | (N_c_ = 128,094)^b^ | |
| Educational Attainment | |  | |  | |  | |  | |  | |  |
| No College Degree | 2,854 (33.6) | | 36,086 (31.7) | | 875 (75.0) | | 10,329 (72.7) | | 3,729 (38.6) | | 46,415 (36.2) | |
| College Degree | 3,081 (36.3) | | 42,715 (37.5) | | 200 (17.2) | | 2,643 (18.6) | | 3,281 (34.0) | | 45,358 (35.4) | |
| Graduate Degree | 2,533 (29.8) | | 34,935 (30.7) | | 84 (7.2) | | 1,204 (8.5) | | 2,617 (27.1) | | 36,139 (28.2) | |
| Missing | 18 (0.2) | | 154 (0.1) | | 7 (0.6) | | 28 (0.2) | | 25 (0.3) | | 182 (0.1) | |
| Marital Status |  | |  | |  | |  | |  | |  | |
| Married | 3,999 (47.1) | | 54,718 (48.0) | | 433 (37.1) | | 5,435 (38.3) | | 4,432 (45.9) | | 60,153 (47.0) | |
| Never married | 2,602 (30.7) | | 35,478 (31.2) | | 372 (31.9) | | 4,271 (30.1) | | 2,974 (30.8) | | 39,749 (31.0) | |
| Widowed, divorced, or separated | 755 (8.9) | | 10,128 (8.9) | | 180 (15.4) | | 2,392 (16.8) | | 935 (9.7) | | 12,520 (9.8) | |
| Part of an unmarried couple | 1,098 (12.9) | | 13,120 (11.5) | | 174 (14.9) | | 2,020 (14.2) | | 1,272 (13.2) | | 15,140 (11.8) | |
| Missing | 32 (0.4) | | 446 (0.4) | | 7 (0.6) | | 86 (0.6) | | 39 (0.4) | | 532 (0.4) | |

Supplementary Table 2. Adjusted change in mean menstrual cycle length and 95% confidence intervals (95% CIs) and adjusted odds ratios (ORs) and 95% CIs of experiencing a long menstrual cycle (> 38 days) comparing vaccinated and unvaccinated participants and cycles in which a vaccine was administered and post-vaccination cycles with pre-vaccination cycles. Differences are from linear mixed-effect regression with participant-specific random intercepts. Odds ratios are from logistic generalized estimating equations. Models are adjusted for race/ethnicity, parity, age, BMI, and seasonality. Doses 1 and 2 were Pfizer-BioNTech or Moderna, and J&J = Johnson & Johnson/Janssen.

|  | Difference in mean cycle length (95% CI) | Odds Ratio of having a long cycle (95% CI) |
| --- | --- | --- |
| Cycles in unvaccinated participants | 0.24 (-0.34, 0.82) | 1.20 (1.00, 1.44) |
| Pre-vaccination cycles | Reference | Reference |
| First dose | 0.61 (0.33, 0.90) | 1.30 (1.11, 1.51) |
| Second dose | 0.54 (0.26, 0.83) | 1.36 (1.16, 1.58) |
| J&J dose | 1.39 (0.57, 2.22) | 2.17 (1.47, 3.19) |
| Post-vaccination cycles |  |  |
| First cycle | 0.43 (0.16, 0.70) | 1.48 (1.28, 1.72) |
| Second cycle | 0.35 (0.08, 0.62) | 1.40 (1.20, 1.63) |
| Third cycle | 0.00 (-0.26, 0.27) | 1.14 (0.98, 1.33) |
| Fourth cycle | -0.17 (-0.44, 0.10) | 0.95 (0.81, 1.11) |

Supplementary Table 3. Adjusted change in mean menstrual cycle length and 95% confidence intervals (95% CIs) comparing vaccinated and unvaccinated participants and cycles in which a vaccine was administered and post-vaccination cycles with pre-vaccination cycles. Differences are from linear mixed-effect regression with participant-specific random intercepts. Differences between regression coefficients are for a given dose in the follicular vs luteal phase. Models are adjusted for race/ethnicity, parity, age, BMI, and seasonality. Doses 1 and 2 were Pfizer-BioNTech or Moderna, and J&J = Johnson & Johnson/Janssen.

|  | Difference in mean cycle length (95% CI) | Difference from coefficient for dose in luteal phase (95% CI) |
| --- | --- | --- |
| Cycles in unvaccinated participants | 0.25 (-0.35, 0.85) | --- |
| Pre-vaccination cycles | Reference | --- |
| Dose in follicular phase |  |  |
| First dose | 1.09 (0.64, 1.54) | 0.77 (0.16, 1.37) |
| Second dose | 1.66 (1.28, 2.03) | 2.57 (1.97, 3.18) |
| J&J dose | 2.42 (1.17, 3.66) | 1.92 (0.21, 3.64) |
| Dose in luteal phase |  |  |
| First dose | 0.32 (-0.03, 0.68) | Reference |
| Second dose | -0.92 (-1.34, -0.49) | Reference |
| J&J dose | 0.50 (-0.66, 1.65) | Reference |
| Post-vaccination cycles |  |  |
| First cycle | 0.46 (0.18, 0.74) | --- |
| Second cycle | 0.37 (0.09, 0.65) | --- |
| Third cycle | 0.03 (-0.25, 0.30) | --- |
| Fourth cycle | -0.15 (-0.43, 0.13) | --- |

Supplementary Table 4. Adjusted within-participant change in mean menstrual cycle length and 95% confidence intervals (95% CIs) comparing cycles in which a vaccine was administered and post-vaccination cycles with pre-vaccination cycles after restricting to participants who tracked at least 3 cycles, restricting to participants with average cycles between 24-38 days, restricting to cycles completed before COVID-19 Vaccine Update survey completion, and restricting to cycles with complete cases. Differences are from conditional linear regression. The conditional model control for all participant-level characteristics; models are additionally adjusted for age, BMI, and seasonality. Doses 1 and 2 were Pfizer-BioNTech or Moderna; J&J = Johnson & Johnson/Janssen.

|  | Participants with 3 or more cycles | Participants with normal average cycles | Cycles before COVID-19 survey completion | Cycles with complete cases |
| --- | --- | --- | --- | --- |
|  | Difference in mean cycle length (95% CI) | Difference in mean cycle length (95% CI) | Difference in mean cycle length (95% CI) | Difference in mean cycle length (95% CI) |
| Pre-vaccination cycles | Reference | Reference | Reference | Reference |
| First dose | 0.52 (0.25, 0.79) | 0.51 (0.26, 0.76) | 0.51 (0.23, 0.79) | 0.49 (0.21, 0.78) |
| Second dose | 0.43 (0.15, 0.71) | 0.40 (0.14, 0.66) | 0.39 (0.10, 0.67) | 0.38 (0.09, 0.67) |
| J&J dose | 1.29 (0.51, 2.08) | 1.23 (0.49, 1.96) | 1.33 (0.52, 2.14) | 1.34 (0.52, 2.17) |
| Post-vaccination cycles |  |  |  |  |
| First cycle | 0.14 (-0.12, 0.41) | 0.11 (-0.15, 0.36) | 0.14 (-0.12, 0.41) | 0.15 (-0.12, 0.43) |
| Second cycle | 0.30 (0.02, 0.57) | 0.33 (0.07, 0.58) | 0.13 (-0.13, 0.40) | 0.15 (-0.12, 0.42) |
| Third cycle | 0.00 (-0.28, 0.28) | 0.00 (-0.26, 0.26) | -0.13 (-0.39, 0.14) | -0.13 (-0.40, 0.15) |
| Fourth cycle | -0.16 (-0.44, 0.13) | -0.06 (-0.32, 0.20) | -0.23 (-0.49, 0.04) | -0.25 (-0.52, 0.02) |

Supplementary Table 5. Adjusted change in mean menstrual cycle length and 95% confidence intervals (95% CIs) comparing vaccinated and unvaccinated participants and cycles in which a vaccine was administered and post-vaccination cycles with pre-vaccination cycles after restricting to participants who tracked at least 3 cycles, restricting to participants with average cycles between 24-38 days, restricting to cycles completed before COVID-19 Vaccine Update survey completion, and restricting to cycles with complete cases. Differences are from linear mixed-effect regression with participant-specific random intercepts. Models are adjusted for race/ethnicity, parity, age, BMI, and seasonality. Doses 1 and 2 were Pfizer-BioNTech or Moderna, and J&J = Johnson & Johnson/Janssen.

|  | Participants with 3 or more cycles | Participants with normal average cycles | Cycles before COVID-19 survey completion | Cycles with complete cases |
| --- | --- | --- | --- | --- |
|  | Difference in mean cycle length (95% CI) | Difference in mean cycle length (95% CI) | Difference in mean cycle length (95% CI) | Difference in mean cycle length (95% CI) |
| Cycles in unvaccinated participants | 1.03 (0.59, 1.48) | 0.18 (-0.12, 0.49) | 0.17 (-0.41, 0.75) | 0.27 (-0.34, 0.88) |
| Pre-vaccination cycles | Reference | Reference | Reference | Reference |
| First dose | 0.58 (0.31, 0.86) | 0.58 (0.32, 0.883) | 0.62 (0.33, 0.90) | 0.61 (0.30, 0.91) |
| Second dose | 0.49 (0.21, 0.78) | 0.47 (0.21, 0.74) | 0.54 (0.25, 0.83) | 0.54 (0.23, 0.85) |
| J&J dose | 1.42 (0.62, 2.22) | 1.34 (0.60, 2.09) | 1.42 (0.59, 2.25) | 1.49 (0.61, 2.36) |
| Post-vaccination cycles |  |  |  |  |
| First cycle | 0.24 (-0.03, 0.52) | 0.21 (-0.05, 0.46) | 0.42 (0.15, 0.69) | 0.45 (0.17, 0.74) |
| Second cycle | 0.38 (0.10, 0.66) | 0.44 (0.18, 0.70) | 0.33 (0.06, 0.60) | 0.37 (0.09, 0.66) |
| Third cycle | 0.06 (-0.23, 0.34) | 0.09 (-0.17, 0.35) | 0.00 (-0.27, 0.27) | 0.04 (-0.24, 0.33) |
| Fourth cycle | -0.12 (-0.41, 0.17) | 0.02 (-0.25, 0.29) | -0.17 (-0.44, 0.10) | -0.16 (-0.45, 0.12) |

Supplementary Table 6. Adjusted within-participant change in mean menstrual cycle length and 95% confidence intervals (95% CIs) comparing cycles in which a vaccine was administered and post-vaccination cycles with pre-vaccination cycles after restricting to participants who reported never testing positive for COVID-19. Differences are from conditional linear regression. The conditional model control for all participant-level characteristics; models are additionally adjusted for age, BMI, and seasonality. Doses 1 and 2 were Pfizer-BioNTech or Moderna; J&J = Johnson & Johnson/Janssen.

|  | Difference in mean cycle length (95% CI) |
| --- | --- |
| Pre-vaccination cycles | Reference |
| First dose | 0.40 (0.04, 0.77) |
| Second dose | 0.36 (-0.02, 0.73) |
| J&J dose | 1.32 (0.25, 2.40) |
| Post-vaccination cycles |  |
| First cycle | 0.30 (-0.06, 0.65) |
| Second cycle | 0.21 (-0.14, 0.56) |
| Third cycle | -0.19 (-0.54, 0.16) |
| Fourth cycle | -0.22 (-0.56, 0.13) |

Supplementary Table 7. Adjusted within-participant change in mean menstrual cycle length and 95% confidence intervals (95% CIs) comparing cycles in which a vaccine was administered and post-vaccination cycles with pre-vaccination cycles, retaining participants who received two doses in a single cycle. Differences are from conditional linear regression. The conditional models control for all participant-level characteristics; models are additionally adjusted for age, BMI, and seasonality. Doses 1 and 2 were Pfizer-BioNTech or Moderna; J&J = Johnson & Johnson/Janssen.

|  | Difference in mean cycle length (95% CI) |
| --- | --- |
| Pre-vaccination cycles | Reference |
| First dose | 0.51 (0.23, 0.79) |
| Second dose | 0.42 (0.13, 0.70) |
| J&J dose | 1.28 (0.46, 2.10) |
| First and second dose | 4.96 (4.42, 5.50) |
| Cycle between doses | -1.94 (-2.65, -1.23) |
| Post-vaccination cycles |  |
| First cycle | 0.18 (-0.07, 0.43) |
| Second cycle | 0.15 (-0.10, 0.41) |
| Third cycle | -0.11 (-0.36, 0.14) |
| Fourth cycle | -0.19 (-0.44, 0.06) |

Supplementary Table 8. Adjusted within-participant change in mean menstrual cycle length and 95% confidence intervals (95% CIs) comparing cycles in which a vaccine was administered and post-vaccination cycles with pre-vaccination cycles by menstrual cycle phase of vaccine dose, retaining participants who received two doses in a single cycle. Differences are from conditional linear regression. The conditional models control for all participant-level characteristics; models are additionally adjusted for age, BMI, and seasonality. Differences between regression coefficients are for a given dose in the follicular vs luteal phase. Doses 1 and 2 were Pfizer-BioNTech or Moderna; J&J = Johnson & Johnson/Janssen.

|  | Difference in mean cycle length (95% CI) | Difference from coefficient for dose in luteal phase (95% CI) |
| --- | --- | --- |
| Pre-vaccination cycles | Reference | --- |
| Dose in follicular phase |  |  |
| First dose | 0.99 (0.55, 1.42) | 0.76 (0.16, 1.36) |
| Second dose | 1.46 (1.09, 1.82) | 2.40 (1.80, 3.00) |
| J&J dose | 2.31 (1.10, 3.51) | 1.92 (0.20, 3.64) |
| First and second dose | 5.02 (4.48, 5.56) | 3.41 (-0.95, 7.77) |
| Dose in luteal phase |  |  |
| First dose | 0.22 (-0.12, 0.57) | Reference |
| Second dose | -0.94 (-1.35, -0.53) | Reference |
| J&J dose | 0.39 (-0.73, 1.51) | Reference |
| First and second dose | 1.61 (-2.54, 5.75) | Reference |
| Post-vaccination cycles |  |  |
| Cycle between doses | -1.92 (-2.62, -1.21) |  |
| First cycle | 0.20 (-0.05, 0.46) | --- |
| Second cycle | 0.18 (-0.08, 0.43) | --- |
| Third cycle | -0.09 (-0.34, 0.16) | --- |
| Fourth cycle | -0.18 (-0.43, 0.08) | --- |

Supplementary Table 9. Apple Women’s Health Survey COVID-19 Vaccine Update survey questions released in September 2021. Master table with surveys, questions, and original source of questions for AWHS Profile, Demographics survey, Menstrual Status survey, Monthly surveys (Menstrual Update, Lactation Update, and Pregnancy Update), Annual Health survey, Quarterly Health survey, Annual Medical History, and Reproductive History available as supplemental material in Mahalingaiah et al, 2021.

|  | Questions | Answer choices | Conditions |
| --- | --- | --- | --- |
| 1 | Did you receive a vaccination for COVID-19? (Select one) | Yes |  |
|  |  | No |  |
|  |  | I prefer not to answer |  |
| 2 | What was the date of your first vaccination? | Date select | If Q1 = "Yes" |
|  |  | I prefer not to answer (exclusive choice) |  |
| 3 | What type of vaccine did you receive? (Select one) | Pfizer-BioNTech | If Q1 = "Yes" |
|  |  | Moderna |  |
|  |  | Johnson & Johnson/Janssen |  |
|  |  | Other |  |
|  |  | I don’t know |  |
|  |  | I prefer not to answer |  |
| 4 | Did you experience any of the following in the 48 hours after your first dose of the vaccine?  (Select all that apply) | Pain at injection site | If Q1 = "Yes" |
|  |  | Redness at injection site |  |
|  |  | Swelling at injection site |  |
|  |  | Tiredness |  |
|  |  | Headache |  |
|  |  | Muscle pain |  |
|  |  | Chills |  |
|  |  | Fever |  |
|  |  | Nausea |  |
|  |  | Other |  |
|  |  | None of the above (exclusive choice) |  |
|  |  | I prefer not to answer (exclusive choice) |  |
| 5 | Did you receive a second vaccination? (Select one) | Yes | If Q1 = "Yes" |
|  |  | No |  |
|  |  | A second vaccine is not required |  |
|  |  | I prefer not to answer |  |
| 6 | What was the date of your second vaccination? | Date select | If Q5 = "Yes" |
|  |  | I prefer not to answer (exclusive choice) |  |
| 7 | What type of vaccine did you receive? (Select one) | Pfizer-BioNTech | If Q5 = "Yes" |
|  |  | Moderna |  |
|  |  | Other |  |
|  |  | I don’t know |  |
|  |  | I prefer not to answer |  |
| 8 | Did you experience any of the following in the 48 hours after your second dose of the vaccine?  (Select one) | Pain at injection site | If Q5 = "Yes" |
|  |  | Redness at injection site |  |
|  |  | Swelling at injection site |  |
|  |  | Tiredness |  |
|  |  | Headache |  |
|  |  | Muscle pain |  |
|  |  | Chills |  |
|  |  | Fever |  |
|  |  | Nausea |  |
|  |  | Other |  |
|  |  | None of the above (exclusive choice) |  |
|  |  | I prefer not to answer (exclusive choice) |  |

**Supplementary Figures**

**Supplementary Figure 1.** Flow chart of study identification, inclusion, and exclusion criteria for current analysis. N_p_ = number of participants. N_c_ = number of cycles.


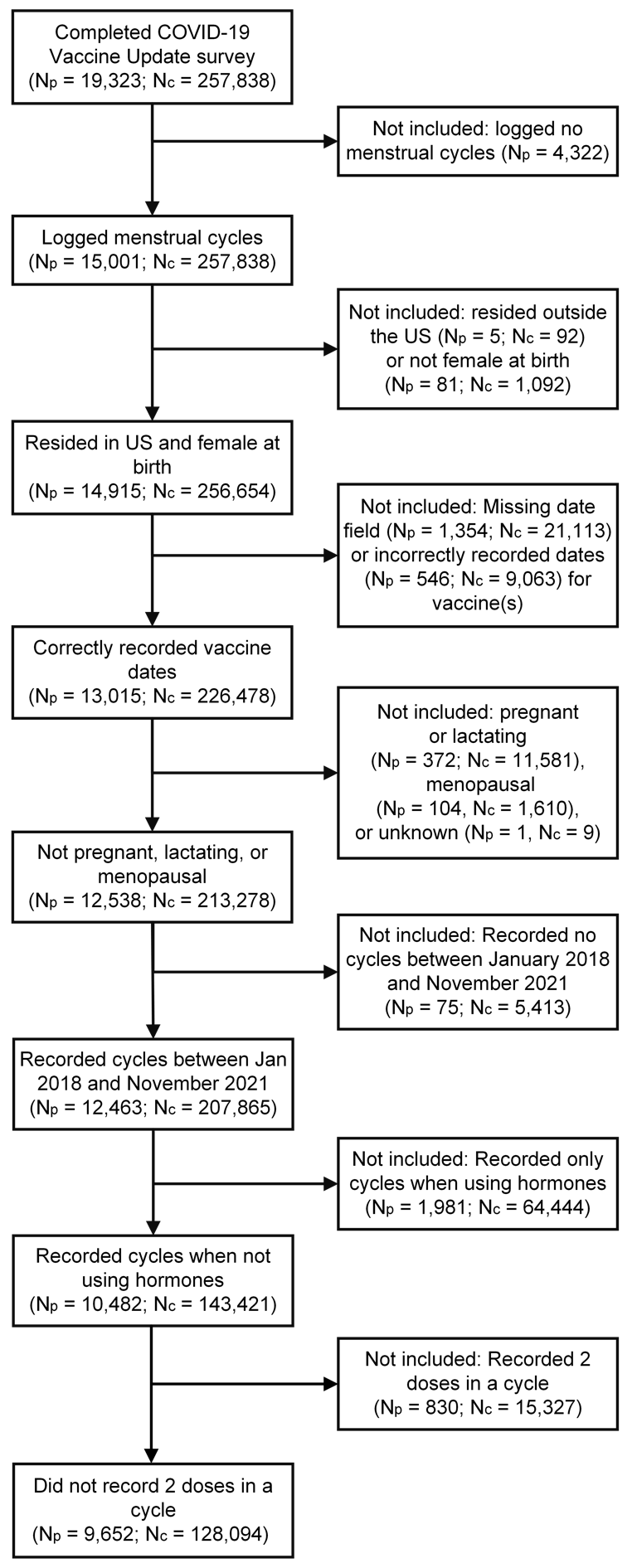


**Supplementary Figure 2.** Histogram of number of cycles reported by participants in the Apple Women’s Health Study.


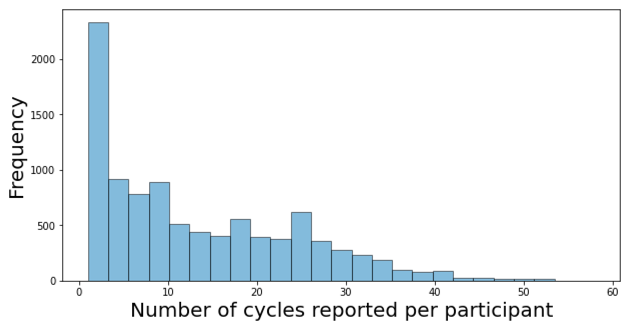


**Supplementary Figure 3.** Adjusted odds ratios (ORs) and 95% confidence intervals (CIs) of experiencing a long menstrual cycle (> 38 days) comparing cycles in which a vaccine was administered and post-vaccination cycles with pre-vaccination cycles. ORs are from conditional logistic regression model that controls for all participant-level characteristics; model additionally adjusted for age, BMI, and seasonality. First and second mRNA doses were Pfizer-BioNTech or Moderna; single dose J&J was Johnson & Johnson/Janssen. ORs are represented by points on the figure, and error bars represent the upper and lower bounds of the 95% CI.

***
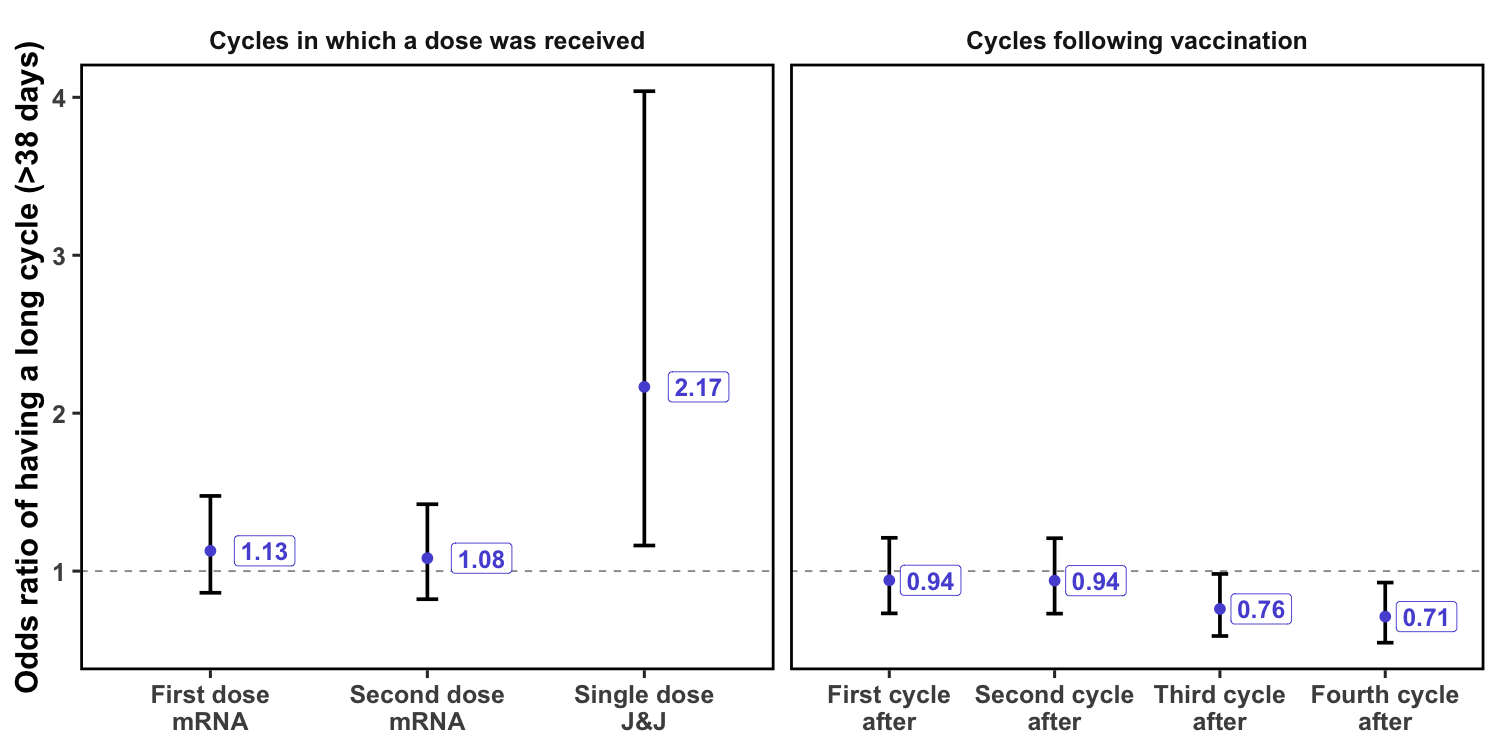
***

**Supplementary Figure 4.** Directed Acyclic Graph (DAG) used to identify confounding variables as covariates for inclusion in regression models; Note: Figure generated using DAGitty v3.0.5. The green variable with the triangle, COVID-19 Vaccine, is the treatment, and the blue variable with the vertical bar, MCL, is the outcome of interest. The green arrow is the association of interest. Variables in red (race/ethnicity, SES, education, age, marital status, BMI, parity, and season) are identified as confounders, and red arrows are confounding paths. The variable in green, region, affects only the exposure, and the black arrow is a non-confounding path. This DAG identifies five variables—age, race/ethnicity, parity, BMI, and season—as the minimally sufficient adjustment set for estimating the total effect of the COVID-19 vaccine on MCL. Acronyms: SES = socioeconomic status; BMI = body mass index; MCL = mean cycle length.


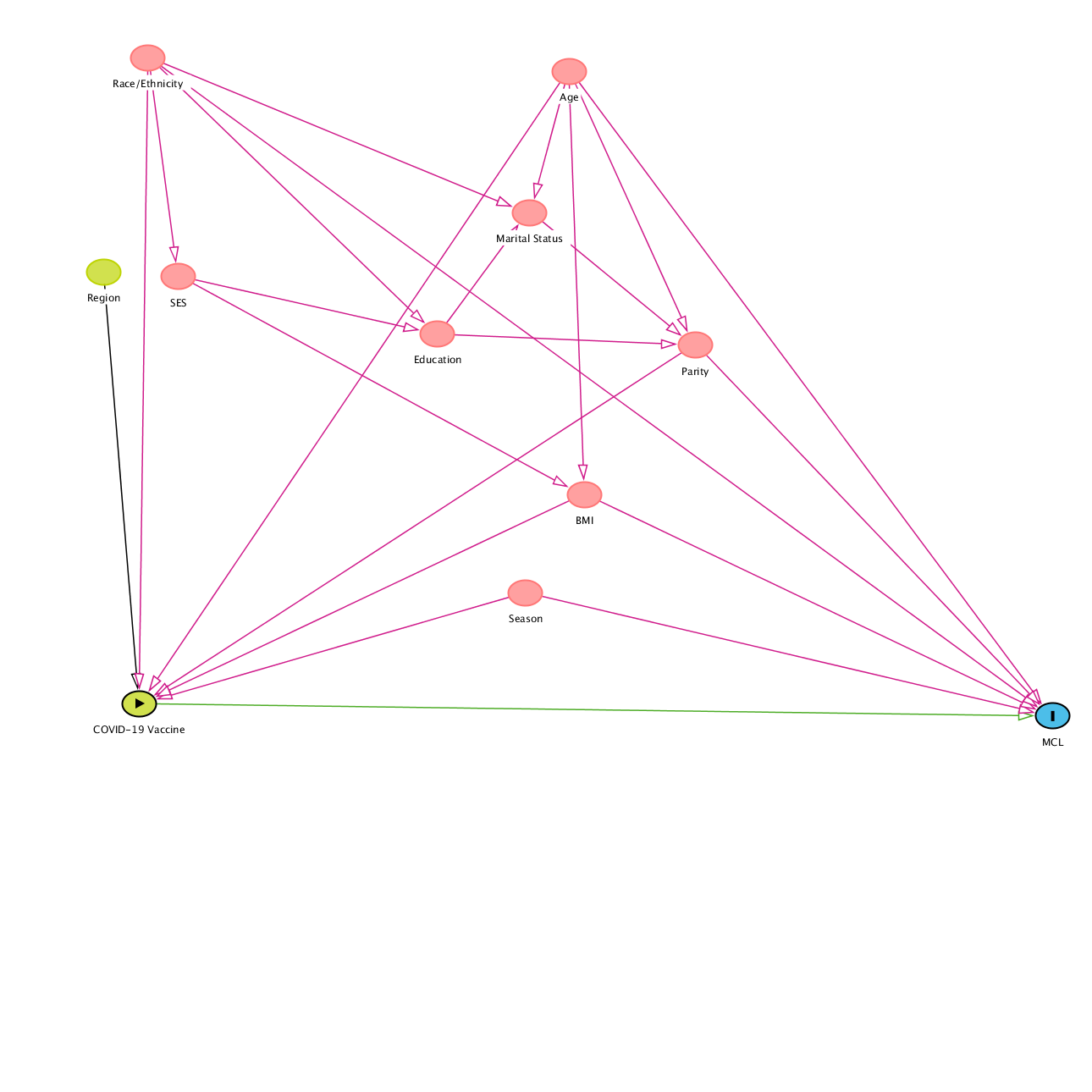

Supplement: Supplementary file 1 — Supplemental material [file 41746_2022_711_MOESM1_ESM.docx]
