# Supplementary material for: Covid-19 vaccination and menstrual cycle length in the Apple Women’s Health Study
Source: NPJ Digit Med. 2022 Nov 2;5:165. doi: 10.1038/s41746-022-00711-9 (PMC9628464; doi:10.1038/s41746-022-00711-9)
Supplement: Supplementary file 2 — Reporting Summary [file 41746_2022_711_MOESM2_ESM.pdf]

nature portfolio

Corresponding author(s): Shruthi Mahalingaiah
Last updated by author(s): Oct 11, 2022

Reporting Summary

Nature Portfolio wishes to improve the reproducibility of the work that we publish. This form provides structure for consistency and transparency in reporting. For further information on Nature Portfolio policies, see our Editorial Policies and the Editorial Policy Checklist.

Statistics

For all statistical analyses, confirm that the following items are present in the figure legend, table legend, main text, or Methods section.

n/a	Confirmed	
☐	☒	The exact sample size (*n*) for each experimental group/condition, given as a discrete number and unit of measurement
☐	☒	A statement on whether measurements were taken from distinct samples or whether the same sample was measured repeatedly
☐	☒	The statistical test(s) used AND whether they are one- or two-sided<br>*Only common tests should be described solely by name; describe more complex techniques in the Methods section.*
☐	☒	A description of all covariates tested
☐	☒	A description of any assumptions or corrections, such as tests of normality and adjustment for multiple comparisons
☐	☒	A full description of the statistical parameters including central tendency (e.g. means) or other basic estimates (e.g. regression coefficient) AND variation (e.g. standard deviation) or associated estimates of uncertainty (e.g. confidence intervals)
☐	☒	For null hypothesis testing, the test statistic (e.g. F, t, r) with confidence intervals, effect sizes, degrees of freedom and P value noted<br>*Give P values as exact values whenever suitable.*
☒	☐	For Bayesian analysis, information on the choice of priors and Markov chain Monte Carlo settings
☐	☒	For hierarchical and complex designs, identification of the appropriate level for tests and full reporting of outcomes
☐	☒	Estimates of effect sizes (e.g. Cohen's *d*, Pearson's *r*), indicating how they were calculated

Our web collection on statistics for biologists contains articles on many of the points above.

Software and code

Policy information about availability of computer code

Data collection	Data management and processing were conducted in Python 3.6.
Data analysis	Statistical analyses were conducted in Python 3.6.

For manuscripts utilizing custom algorithms or software that are central to the research but not yet described in published literature, software must be made available to editors and reviewers. We strongly encourage code deposition in a community repository (e.g. GitHub). See the Nature Portfolio guidelines for submitting code & software for further information.

Data

Policy information about availability of data

All manuscripts must include a data availability statement. This statement should provide the following information, where applicable:

- Accession codes, unique identifiers, or web links for publicly available datasets
- A description of any restrictions on data availability
- For clinical datasets or third party data, please ensure that the statement adheres to our policy

Aggregated data that support the findings of this study may be available upon request from the corresponding author [SM]. Any request for data will be evaluated and responded to in a manner consistent with policies intended to protect participant confidentiality and language in the Study protocol and informed consent form.

Human research participants

Policy information about studies involving human research participants and Sex and Gender in Research.

Reporting on sex and gender	Findings apply only to people who were assigned female at birth. Sex at birth was determined based on self-report. Sex was considered in study design. Participants were only included if they reported that they had been assigned female at birth and were currently menstruating.
Population characteristics	See below.
Recruitment	The following is an excerpt from Mahalingaiah, et al., which detailed recruitment and marketing.<br><br>"On September 10, 2019, the Apple Research app was announced publicly. The Research app houses the following 3 research studies: the AWHS, the Apple Heart and Movement Study, and the Apple Hearing Study. The AWHS was launched on November 14, 2019. Institutional review board-approved recruitment efforts included a Harvard T.H. Chan School of Public Health study website, which was made public and includes frequently asked questions and background information regarding the study. Social media accounts were created on Twitter and Instagram (March 10, 2020), YouTube (March 12, 2020), Facebook (April 13, 2020) and LinkedIn (August 7, 2020) to post recruitment materials inviting potential participants to join the study, with new posts added approximately 1 to 3 times per week. In addition, media events included a podcast, media articles, and press interviews after study launch."<br><br>Mahalingaiah, S., Fruh, V., Rodriguez, E., Konanki, S. C., Onnela, J. P., de Figueiredo Veiga, A., ... & Williams, M. A. (2022). Design and methods of the Apple Women's Health Study: a digital longitudinal cohort study. American Journal of Obstetrics and Gynecology, 226(4), 545-e1.<br><br>Because our study population only included iPhone users in the U.S. and our recruitment specifically targeted social media users, our results may not be generalizable to all U.S. individuals who menstruate or to other populations.
Ethics oversight	This study was approved by the Institutional Review Board at Advarra (CIRB #PRO00037562) and registered on Clinicaltrials.gov (NCT04196595).

Note that full information on the approval of the study protocol must also be provided in the manuscript.

Field-specific reporting

Please select the one below that is the best fit for your research. If you are not sure, read the appropriate sections before making your selection.

☐ Life sciences ☒ Behavioural & social sciences ☐ Ecological, evolutionary & environmental sciences

For a reference copy of the document with all sections, see nature.com/documents/nr-reporting-summary-flat.pdf

Behavioural & social sciences study design

All studies must disclose on these points even when the disclosure is negative.

Study description	This is a quantitative prospective cohort study.
Research sample	The research sample is people assigned female at birth who have menstruated at least once, live in the U.S., are at least 18 years old (at least 19 years old in Alabama and Nebraska and at least 21 years old in Puerto Rico), are able to communicate through written and spoken English, have an iPhone with a compatible version of iOS, downloaded the Apple Research app, and are the sole user of an iPhone and an iCloud account.<br><br>Eighty-eight percent of participants were vaccinated; 12% were not. Fifty-five percent of vaccinated participants received the Pfizer-BioNTech vaccine (N = 4,265), 37% received Moderna (N = 3,129), and 8% received J&J (N = 676). For cycles in which a dose was received, 3,410 participants received dose 1 (2.7% of all cycles), 3,454 received dose 2 (2.7%), and 337 received the J&J dose (0.3%). A total of 7,490 long cycles were identified, 6% of all cycles included. Vaccinated participants tended to be in the older age groups [N = 2,526 (30%) vaccinated > 40 years old, N = 242 (21%) unvaccinated > 40 years old], to have a college or graduate degree [N = 5,614 (66%) vaccinated, N = 284 (24%) unvaccinated], to be married [N = 3,999 (47%) vaccinated, N = 433 (37%) unvaccinated], and to be nulliparous [N = 5,101 (60%) vaccinated, N = 512 (44%) unvaccinated (Table 1)]. The distribution of BMI and race/ethnicity were similar between vaccinated and unvaccinated participants.
Sampling strategy	This study was conducted within an existing cohort, and no sampling was performed for this study.
Data collection	This was a mobile-application-based longitudinal cohort study involving survey data. The study is hosted within the Apple Research app (available on the Apple App Store), which allows a participant to find, enroll, and participate in Apple-supported, health-related research studies. Participants respond to surveys through their phones, not in the presence of the research team.

	On enrollment, participants responded to the Research Profile Survey within the Research app and to the Demographic Survey; the data included the year of birth, state of residence, race and ethnicity, marital status, employment status, gender identity, and sex assigned at birth. Participants were asked to respond to a baseline Menstrual Status Survey at enrollment. After enrollment, participants were given monthly surveys to provide a menstrual status update. Participants logged menses, with associated dates, via Cycle Tracking in the Apple Health app or any third-party cycle tracking app that the participant permitted to share data as part of this study.
Timing	Recruitment began in November 2019 and is currently on-going; the last recruitment date for this cohort was January 31, 2022. The primary analysis included cycles tracked retrospectively as early as January 2018, and prospectively between enrollment and November 2021.
Data exclusions	For this analysis, we included eligible AWHS participants who completed the COVID-19 Vaccine Update survey and tracked at least one menstrual cycle. Cycles were excluded if participants reported a hysterectomy or were pregnant, lactating, menopausal, or using hormones. Exclusion criteria were pre established. Exclusion criteria were conditions that prevent menstruation or that control menstruation in such a way that would interfere with the treatment of interest.
Non-participation	Of 19,323 participants who completed the COVID-19 Vaccine Update survey, 4,322 logged menstrual cycles (77.6% response rate).
Randomization	Participants were not allocated into experimental groups.

Reporting for specific materials, systems and methods

We require information from authors about some types of materials, experimental systems and methods used in many studies. Here, indicate whether each material, system or method listed is relevant to your study. If you are not sure if a list item applies to your research, read the appropriate section before selecting a response.

Materials & experimental systems

n/a	Involved in the study
☒	☐ Antibodies
☒	☐ Eukaryotic cell lines
☒	☐ Palaeontology and archaeology
☒	☐ Animals and other organisms
☒	☐ Clinical data
☒	☐ Dual use research of concern

Methods

n/a	Involved in the study
☒	☐ ChIP-seq
☒	☐ Flow cytometry
☒	☐ MRI-based neuroimaging
